# Exceptional preservation of internal organs in a new fossil species of freshwater shrimp (Caridea: Palaemonoidea) from the Eocene of Messel (Germany)

**DOI:** 10.1038/s41598-022-23125-9

**Published:** 2022-10-27

**Authors:** Valentin de Mazancourt, Torsten Wappler, Sonja Wedmann

**Affiliations:** 1 Center for Integrative Biodiversity Discovery, Museum für Naturkunde Leibniz Institute for Evolution and Biodiversity Science, Berlin, Germany; 2 Laboratoire Biologie des Organismes et Écosystèmes Aquatiques MNHN, CNRS 8067, SU, IRD 207, UCN, UA, Sorbonne Université, Paris, France; 3 Department of Natural History, Hessisches Landesmuseum Darmstadt, Darmstadt, Germany; 4 Section Palaeontology, Institute of Geosciences, Rheinische Friedrich-Wilhelms-Universität Bonn, Bonn, Germany; 5 Senckenberg Forschungsstation Grube Messel, Senckenberg Forschungsinstitut und Naturmuseum Frankfurt/M., Messel, Germany

**Keywords:** Zoology, Palaeoecology, Palaeontology, Taxonomy

## Abstract

A new species of extinct freshwater shrimp was discovered in the Eocene deposit of the Messel Pit Konservat-Lagerstätte. This rare find is represented by only a few specimens, one of which showing exceptionally preserved soft tissues and other internal parts like the stomach with possibly gastric ossicles in place, branchiae, the ovary, and the left mandible, never described in a fossil shrimp. The new species *Bechleja brevirostris* n. sp. is characterized by a short rostrum bearing 6–8 dorsal spines and one ventral tooth, and long second pereiopods with strong chelae. One additional specimen shows a slightly different morphology and might belong to a different species. The systematic position of the species among the superfamily Palaemonoidea is discussed, as well as implications for the knowledge of the paleoenvironment of Lake Messel and the paleobiogeography of the Eocene.

## Introduction

Caridean shrimps are decapod crustaceans characterized by a long laterally compressed abdomen and chelae on their first two pairs of pereiopods. They are currently represented by more than 3400 species found worldwide in a variety of aquatic habitats, from the deep sea to inland freshwaters^1^. Shrimps of the infraorder Caridea are notably scarce in the fossil record with only around 50 species described^1^. The fossils are often not well-preserved^2,3^, which makes their systematic placement rather difficult and their use for studying evolution risky.

From the Eocene Fossillagerstätte Grube Messel in Germany, only few fossil freshwater shrimps were found of which especially one fossil shows exquisite soft tissue preservation. The Messel Pit Fossil Site is a UNESCO world heritage site which is renowned for exceptional soft tissue preservation of fur and feathers (e.g.,^4,5^), but also of structural colors in insects (e.g.,^6^).

Already Rietschel^7^ reported the presence of undetermined fossil shrimps from Messel, and later, shrimps were pictured by Wolf^8^, Rabenstein^9^ and Gruber & Micklich^10^. Wedmann^11^ indicated in her checklist of invertebrate fossils from Messel the occurrence of shrimps identified as belonging to the family Atyidae by M. Türkay (Senckenberg Museum) in 2003, but no further results have been published on their account until now.

The aim of the present article is to report on the discovery of a new species of freshwater shrimp with exceptionally preserved internal organs and formally describing it.

### Geology and palaeontology of Messel (Fig. 1)

**Figure 1:**
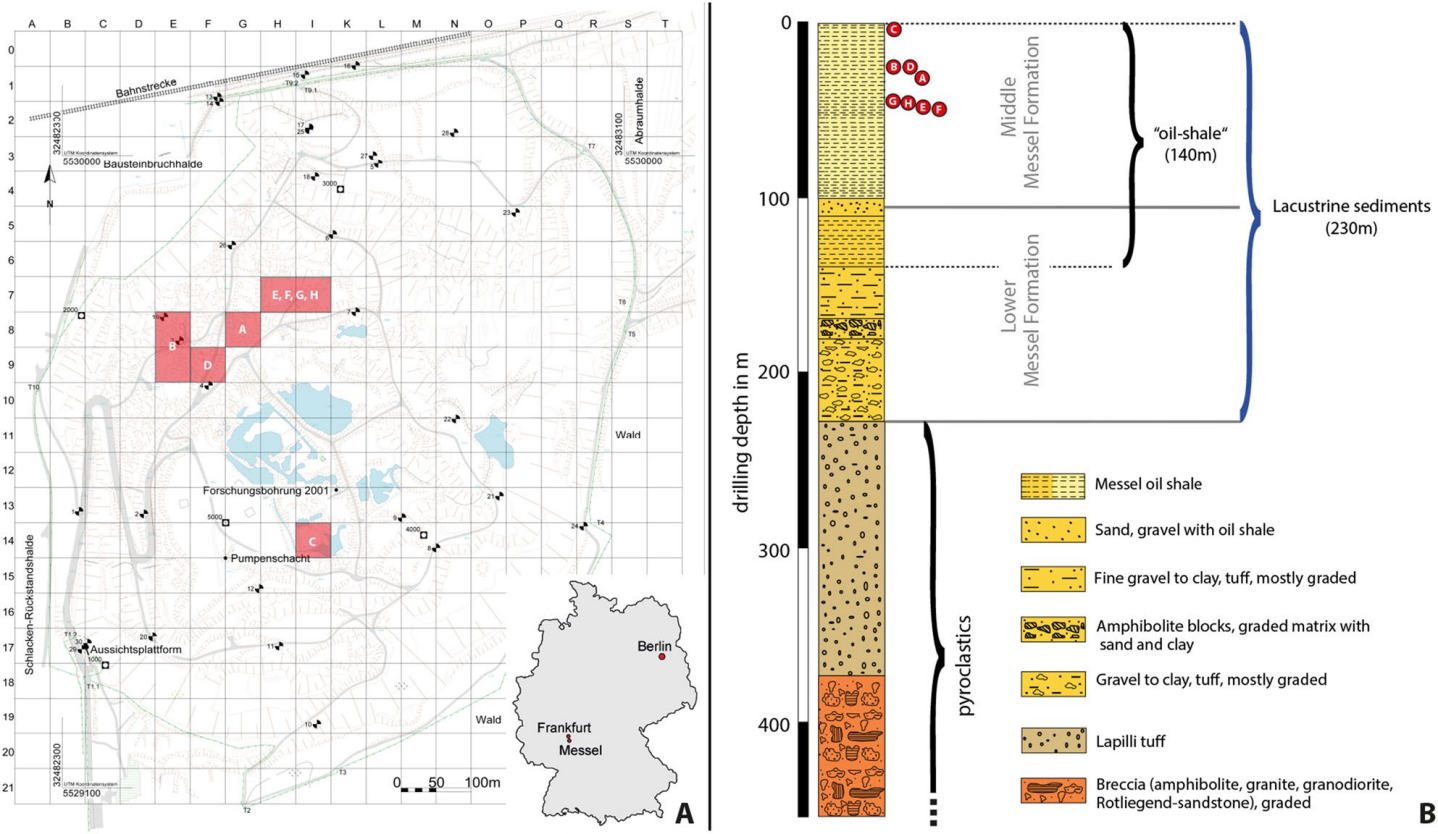
(**A**) Map of the Grube Messel site (from^12^) with the location of each shrimp fossil (A–H) indicated. (A) SF-MeI 5933 (holotype); (B) SF-MeI 13611; (C) SF-MeI 14640; (D) SF-MeI 16018; (E) HLMD-Me-10684; (F) HLMD-Me-10646; (G) HLMD-Me-13919 and (H) HLMD-Me-13920. (**B**) Section of the Grube Messel core (modified from^13^). Red circles indicate the corresponding layers where the fossil shrimps were found. Depth: for (C) ca. 2.96 m to 2.16 m; for (B) and (D): ca. 24.86 m to 23.86 m; for (A): ca. 27.46 m to 27.06 m; for (G): ca. 46.49 m to 45.97 m; for (H): ca. 46.03 m; for (E): ca. 46.26 m; for (F): ca. 47.2 m.

The UNESCO world heritage Messel pit fossil site is located on the “Sprendlinger Horst”, an uplifted Palaeozoic basement block, about 30 km south of the city Frankfurt/M. in the state of Hesse, Germany (Fig. 1A). Volcanic activity during the Eocene created a maar lake which filled with sedimentary deposits^14–16^. In its early phase, the former maar lake was holomictic, but after the stabilization of the crater walls, Lake Messel became permanently meromictic with anoxic bottom water in its deep part, the monimolimnion^17–19^. The sediments of the monimolimnion were transformed into the so-called ‘oil-shale’, a thick sequence of mostly laminated, bituminous and water-rich black pelites^15,19^ (Fig. 1B). The age of the maar formation was ^40^Ar/^39^Ar dated as around 48.2 million years^20,21^ with the Ypresian/Lutetian boundary (currently at 47.8 million years) lying between 30 and 59 m depth in the Research drilling core 2001^21,22^. The upper 90 m of laminated oil shale from that core have sedimentation rates of in average 0.14 mm per year which suggests that Lake Messel existed more than 640,000 years^23^, other estimates go up to one million years^17^. When Lake Messel existed, the climate was paratropical, humid and warm with some seasonality^24^. Mean annual temperatures were reconstructed to have been between 16.8 and 23.9 °C via analysis of plants^25^, and measurements of isotopes in vertebrates led to similar values of around ~ 18 ± 2.5 °C^26^. In the area of the Messel maar lake, paratropical forests were dominant. The vegetation was mainly subtropical to tropical, but also contained plant taxa that are found today in temperate climates^24,27^. An actual overview of the diverse flora and fauna preserved in the oil-shale of Messel is given in Smith et al.^28^. Found macrofossils comprise a high diversity of plants (e.g.,^27^) and also highly diverse insects and other invertebrates (e.g.,^29,30^). Among the vertebrates, especially the mammals are famous, as they allow a view into the early evolution of this group. Iconic groups comprise e.g. the small primeval horses (e.g.^31,32^) or primates like ‘Ida’ (e.g.,^33,34^).

## Results

### Systematic paleontology

**Order Decapoda Latreille, 1802**

**Infraorder Caridea Dana, 1852**

**Superfamily Palaemonoidea Rafinesque, 1815**

**? Family Palaemonidae Rafinesque, 1815**

**Genus** ***Bechleja*** **Houša, 1957**

*Type species: Bechleja inopinata* Houša, 1957 (Oligocene of Czech Republic)^35^.

*Diagnosis:* Rostrum serrate, first pereiopod with a small chela, second pereiopod with a large chela, third to fifth pereiopods sub-equal, small telson, shorter than uropods, antennae 1.5 times as long as cephalothorax^35,36^.

***Bechleja brevirostris* n. sp.** (Figs. 2, 3, 4, 5, 6, 7, 8 and 9).

**Figure 2:**
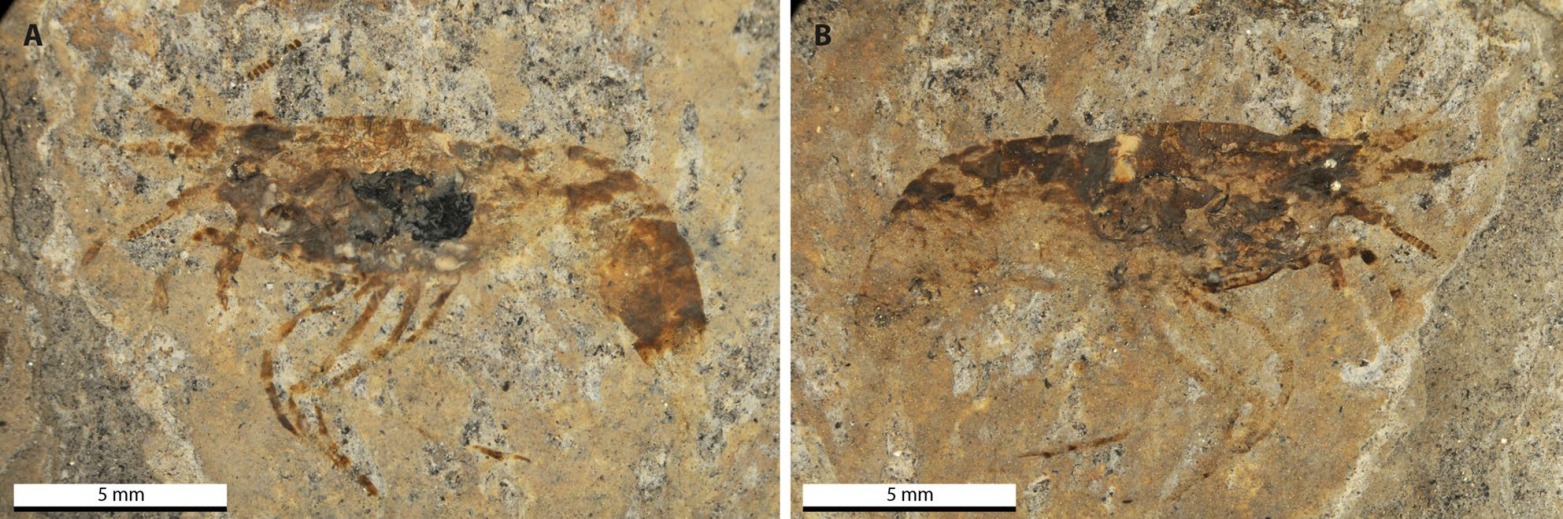
Photograph of the holotype of *Bechleja brevirostris* n. sp. (SF-MeI 5933). (**A**) Plate; (**B**) counterplate.

**Figure 3:**
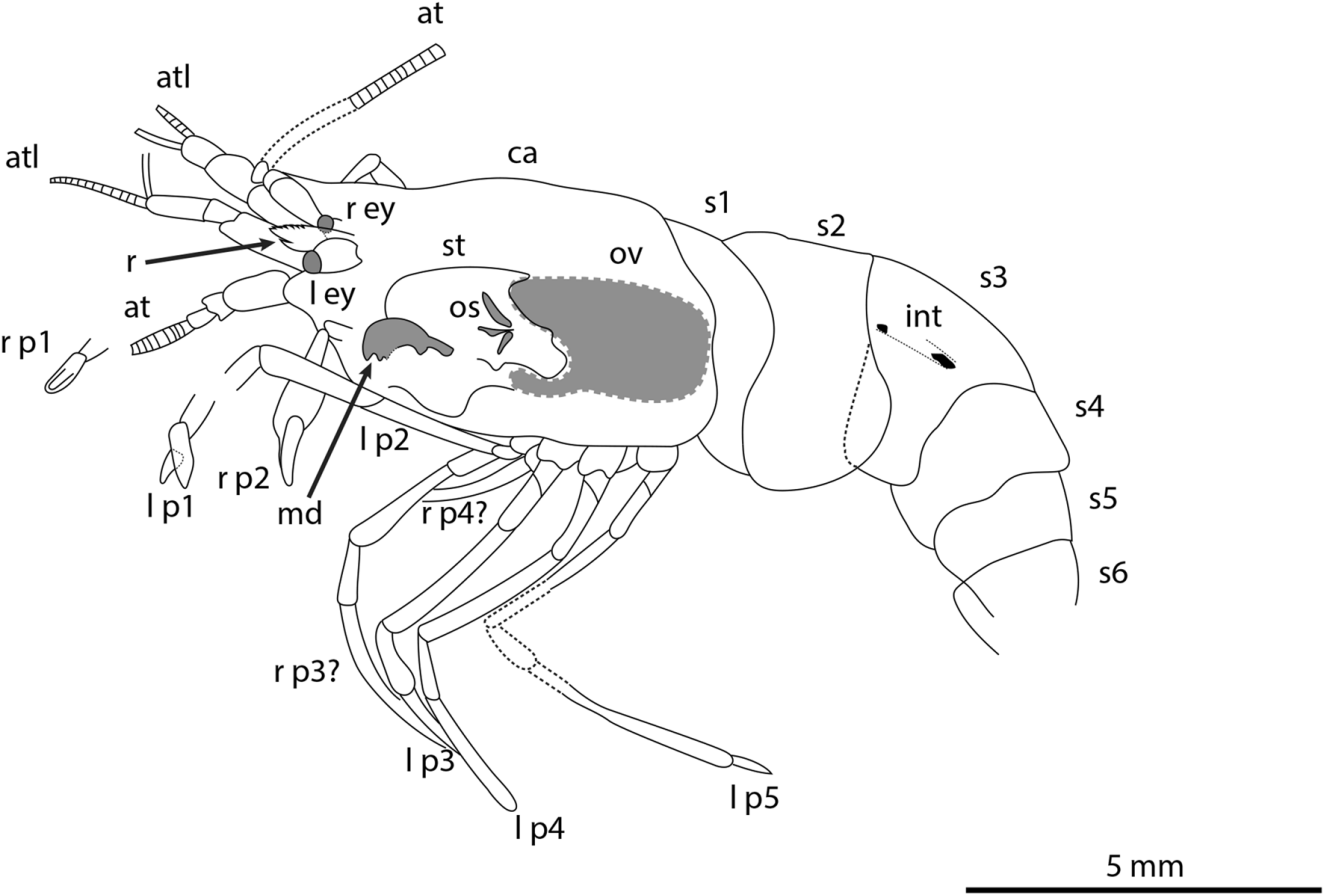
Interpretative drawing of the holotype of *Bechleja brevirostris* n. sp. based on both sides of the fossil (SF-MeI 5933 A and B). atl, antennules; at, antenna; r, rostrum; r ey, right eye; l ey, left eye; ca, carapace; r p1, right first pereiopod; l p1, left first pereiopod; r p2, right second pereiopod; r p3, right third pereiopod; l p3, left third pereiopod; r p4, right fourth pereiopod; l p4, left fourth pereiopod; l p5, left fifth pereiopod; md, left mandible; st, stomach; os, gastric ossicles; ov, ovary; int, intestine; s1, first abdominal somite; s2 second abdominal somite; s3, third abdominal somite; s4, fourth abdominal somite; s5, fifth abdominal somite; s6, sixth abdominal somite.

**Figure 4:**
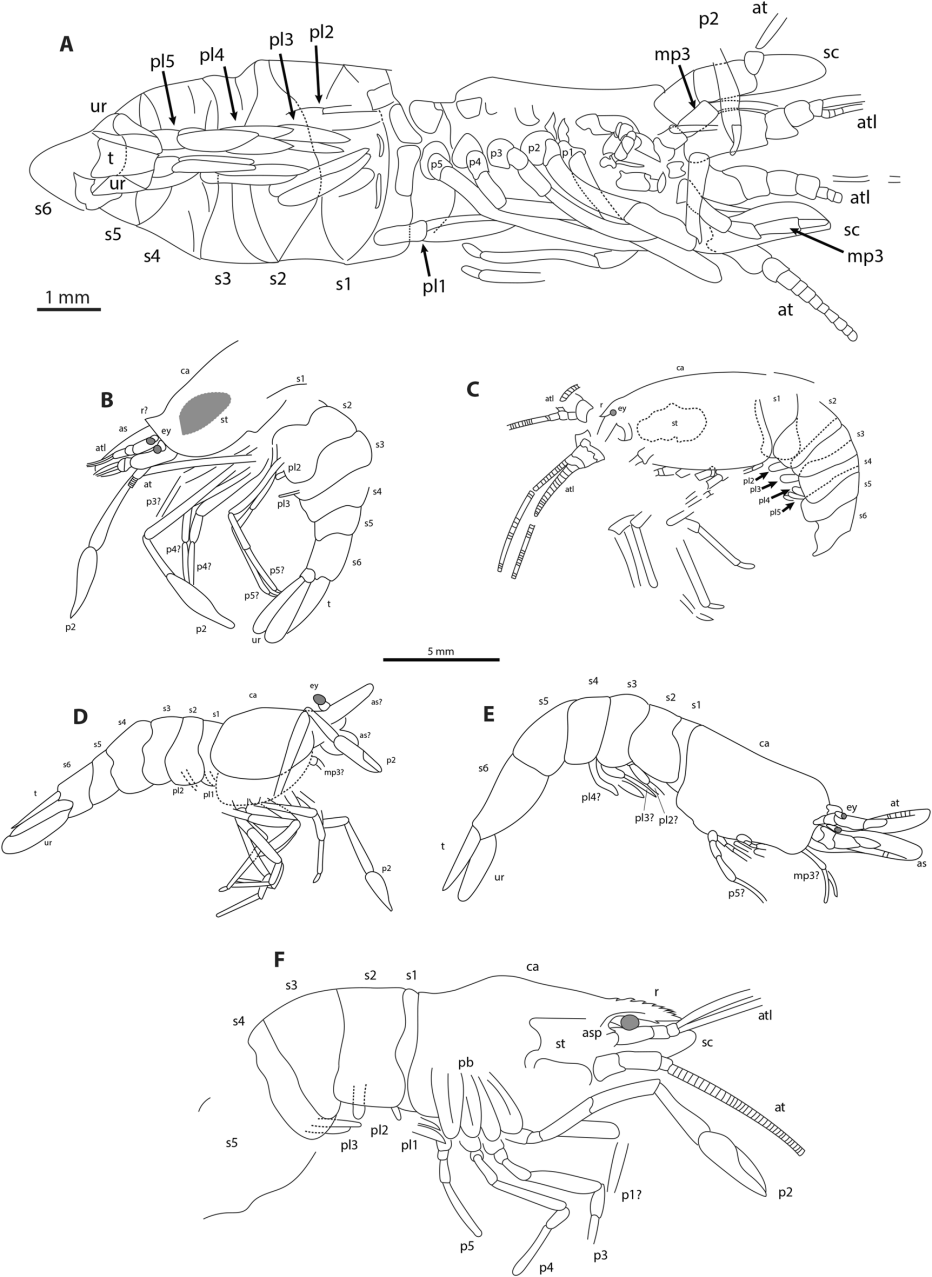
Interpretative drawings of the paratypes of *Bechleja brevirostris* n. sp. based on both sides of the fossils. (**A**) HLMD-Me 10684; (**B**) SF-MeI 13611; (**C**) HLMD-Me 13920; (**D**) SF-MeI 14640; (**E**) SF-MeI 16018; (**F**) HLMD-Me 13919. Abbreviations of morphological characters same as the Fig. 3 with the addition of as, antennal scale/scaphocerite; asp, antennal spine; mp3, third maxilliped; pb, pleurobranchiae; pl1, first pleopod; pl2, second pleopod; pl3, third pleopod; pl4, fourth pleopod; t, telson; ur, uropod. B-F at the same scale.

**Figure 5:**
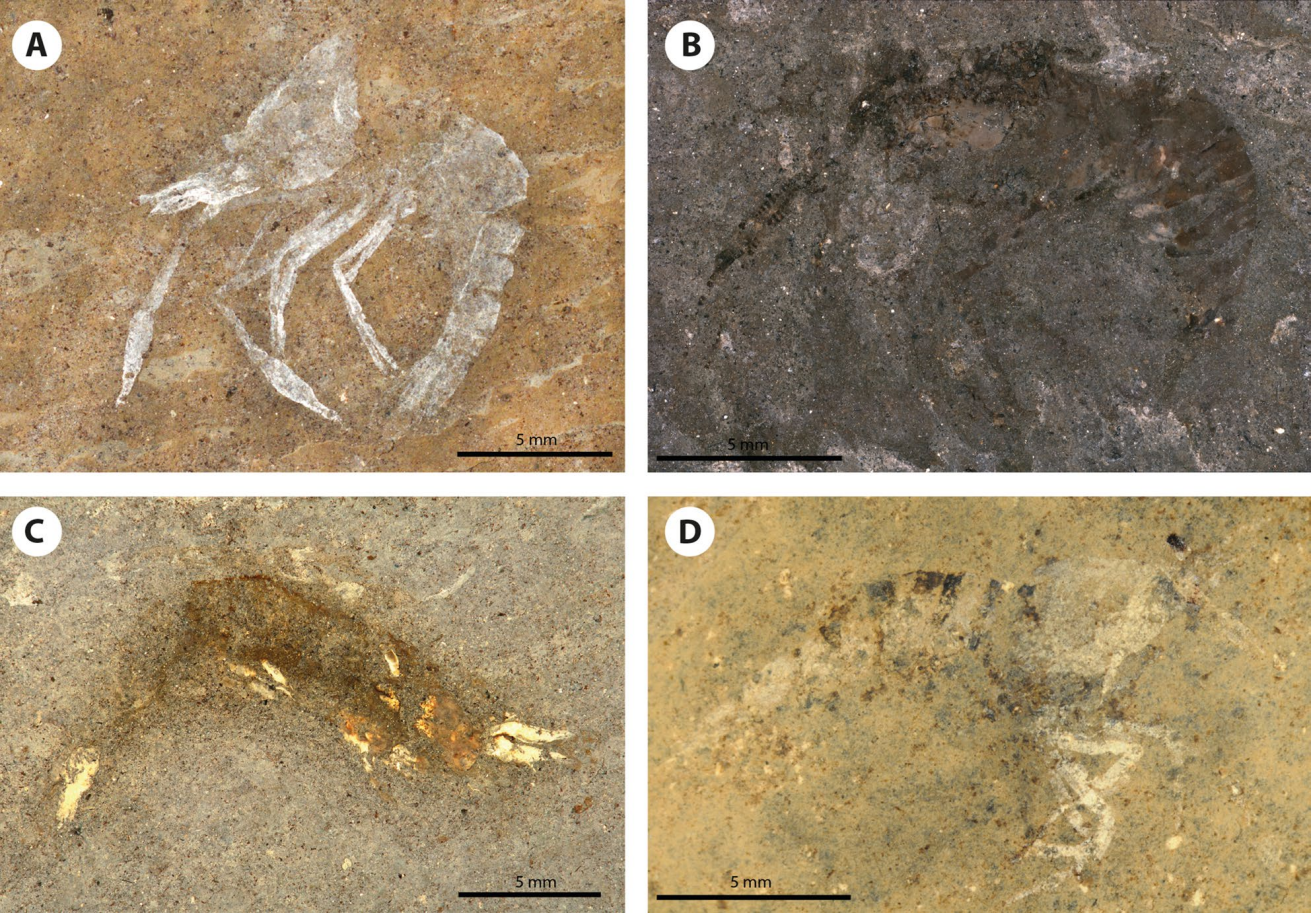
Photographs of the paratypes of *Bechleja brevirostris* n. sp. (**A**) SF-MeI 13611 A; (**B**) HLMD-Me 13920; (**C**) SF-MeI 16018 A; (**D**) SF-MeI 14640 A.

**Figure 6:**
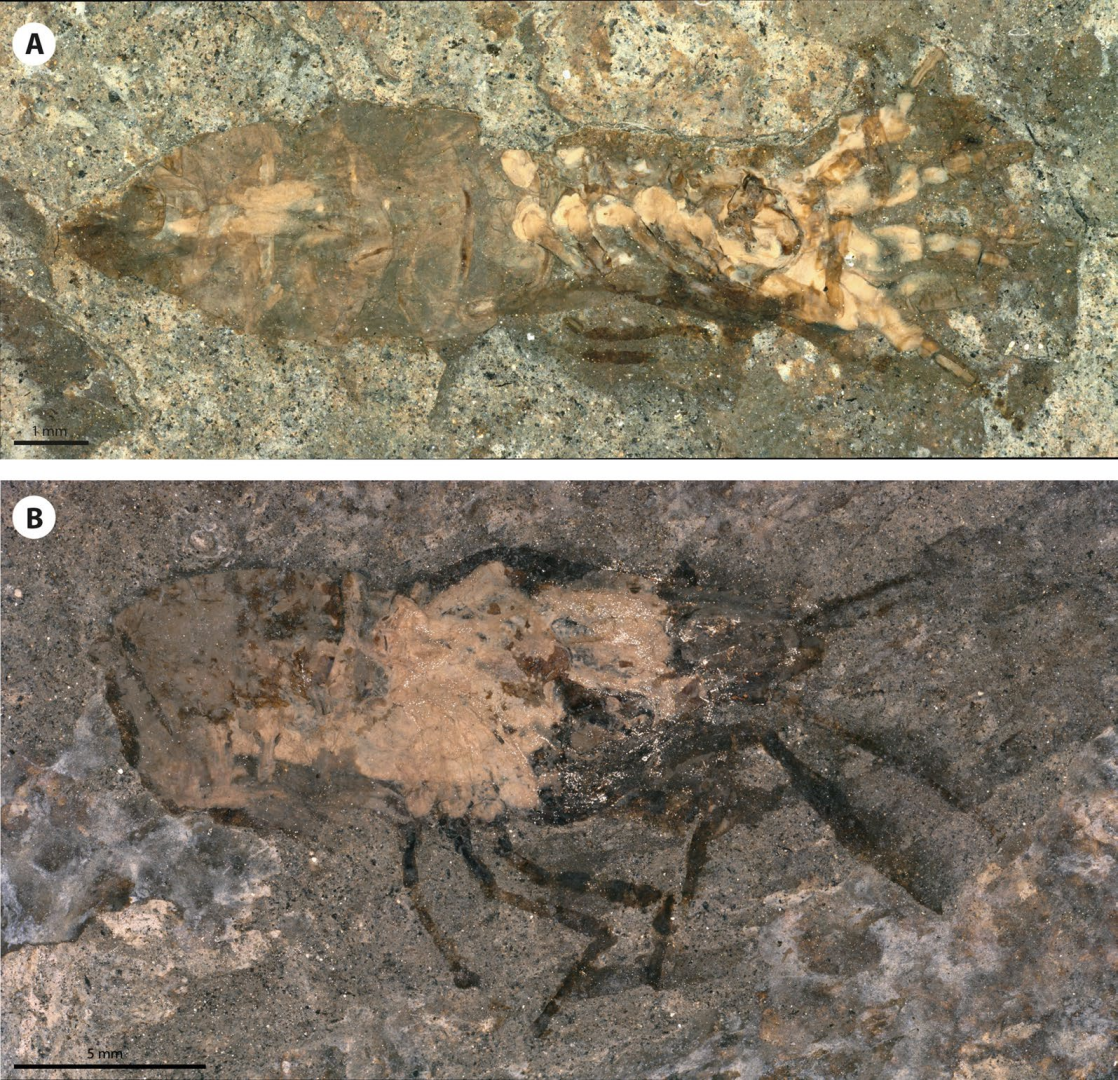
Photographs of the paratypes of *Bechleja brevirostris* n. sp. (**A**) HLMD-Me 10684; (**B**) HLMD-Me 13919.

**Figure 7:**
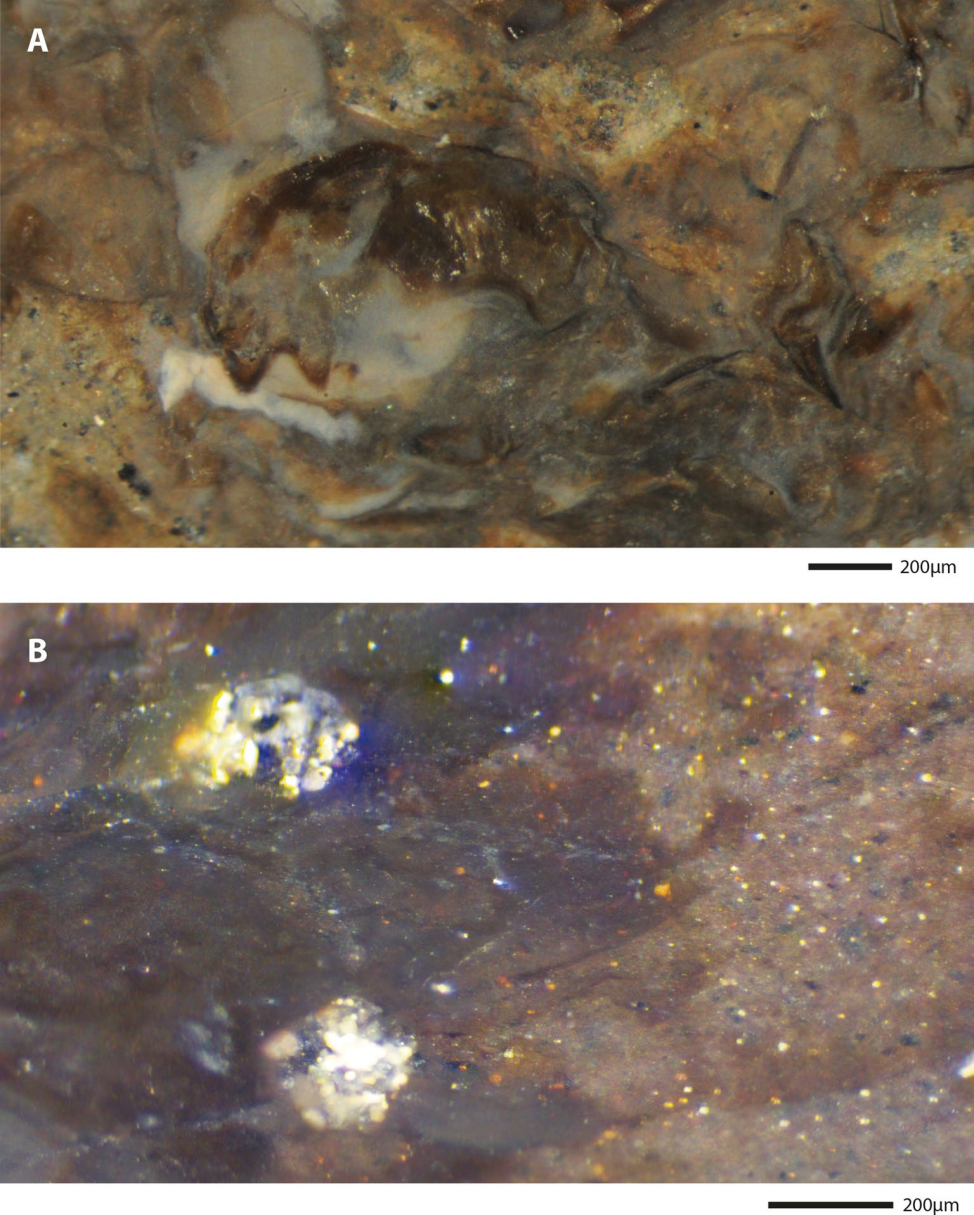
Detail photographs of the holotype *Bechleja brevirostris* n. sp. (**A**) View of the left mandible (SF-MeI 5933 A); (**B**) View of the rostrum and eyes (SF-MeI 5933 B).

**Figure 8:**
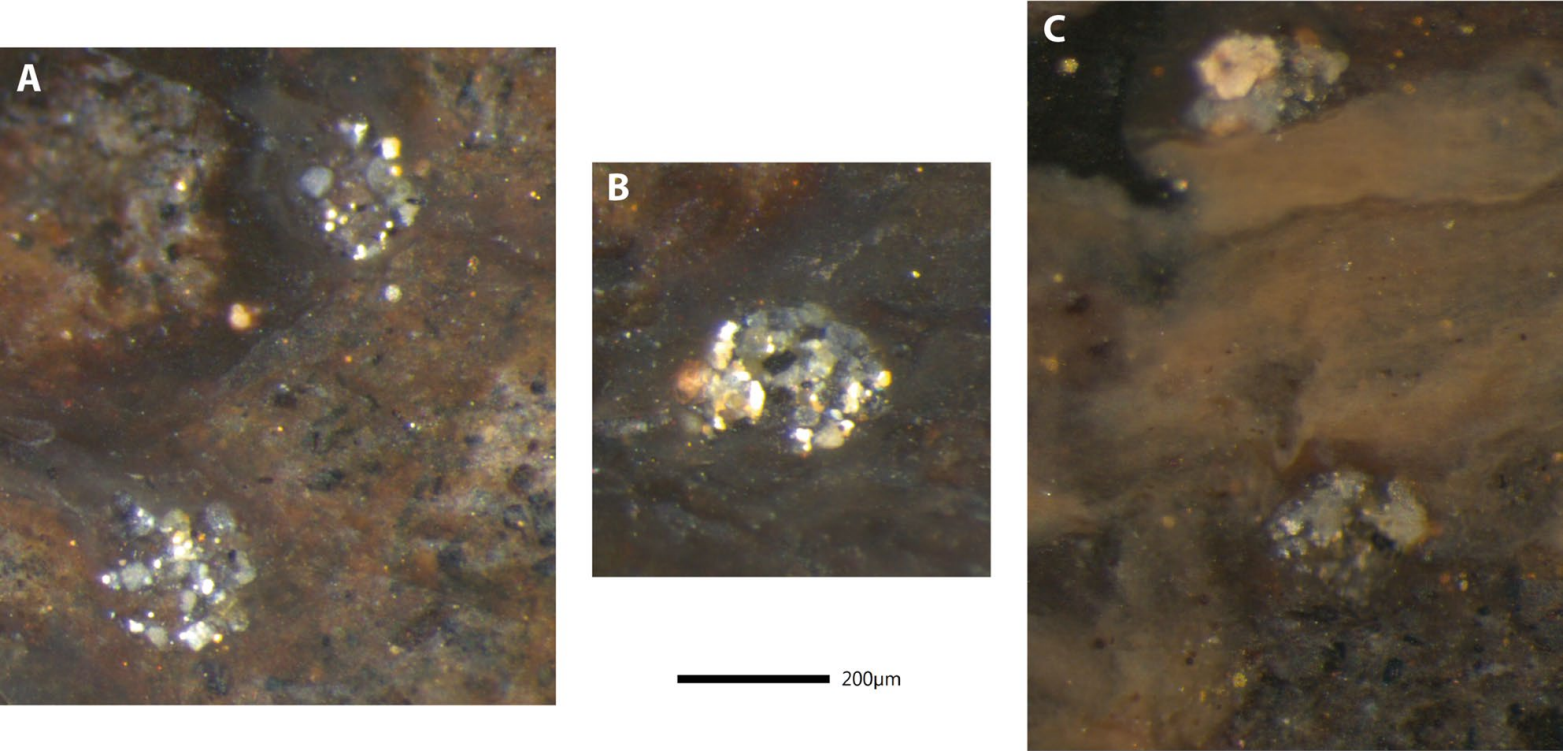
Zoomed photographs of the eyes of two specimens. (**A**) SF-MeI 5933 A; (**B**) SF-MeI 5933 B; (**C**) SF-MeI 16018 B.

**Figure 9:**
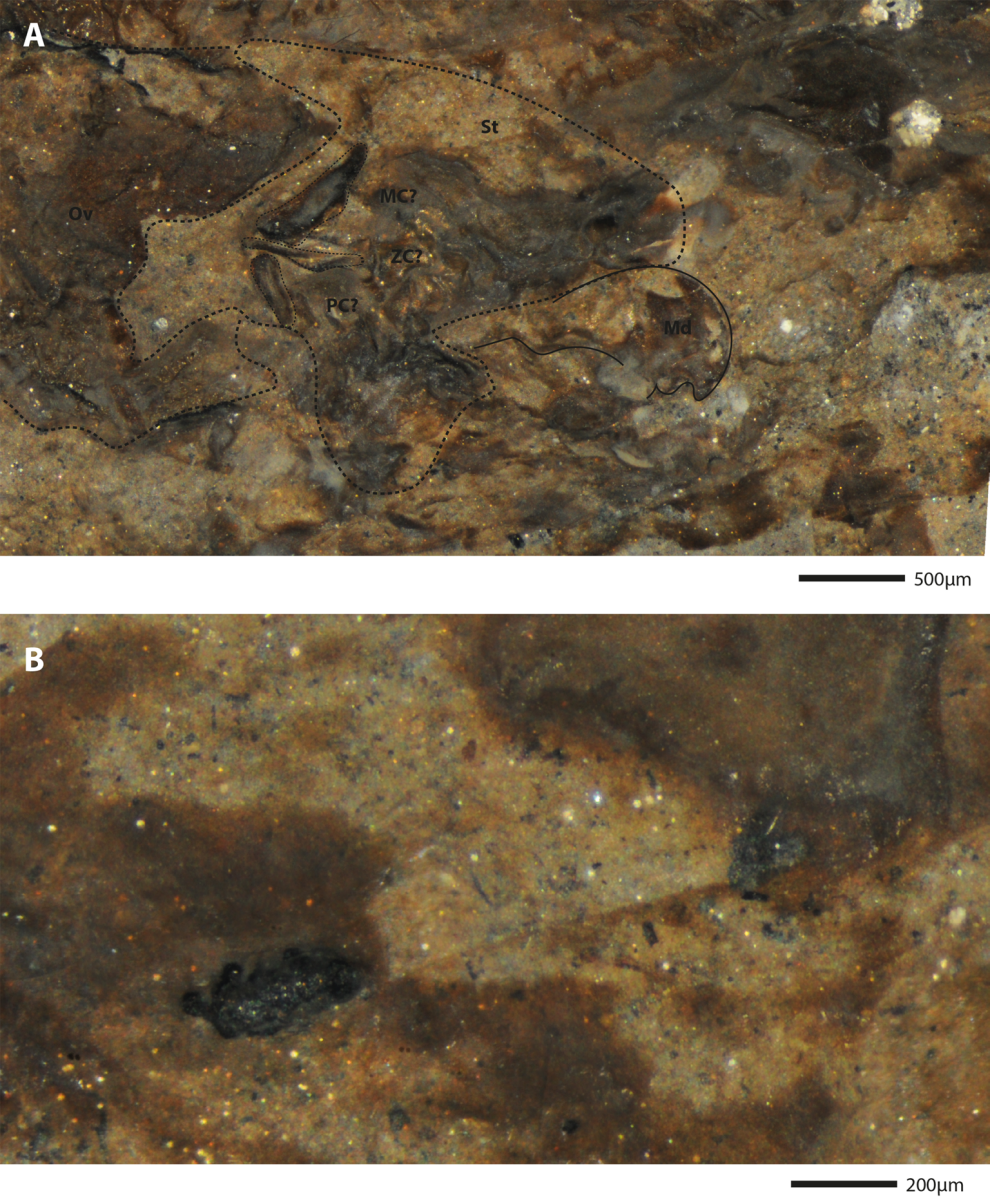
Zoomed photographs of the holotype *Bechleja brevirostris* n. sp. (SF-MeI 5933 B). (**A**) View of the stomach: St, stomach; Md, mandible; MC, mesocardiac ossicle; ZC, zygocardiac ossicle; PC, pterocardiac ossicle; Ov, ovary. (**B**) View of the preserved section of intestine with two dark fecal pellets.

*Type material:* SF-MeI 5933, holotype, plate (A) and counterplate (B); SF-MeI 13611, plate (A) and counterplate (B); SF-MeI14640, plate (A) and counterplate (B), SF-MeI 16018, plate (A) and counterplate (B), HLMD-Me 10684, HLMD-Me 13919, HLMD-Me 13920, paratypes.

*Type locality*: Grube Messel, near Darmstadt, Hesse, Germany (Fig. 1).

*Stratigraphic information*: Holotype SF-MeI 5933: grid square G8; 0.1 m below to 0.3 m above local stratigraphic marker level alpha; SF-MeI 13611: grid square E8/9; 2.5 m above to 3.5 m above local stratigraphic marker level alpha; SF-MeI 14640: grid square i14; 0.95 m above to 1.75 m above local stratigraphic marker level M; SF-MeI 16018: grid square F9; 2.5 m above to 3.5 m above local stratigraphic marker level alpha; HLMD-Me-10684: grid square H/I7; 1.86 m below stratigraphic marker gamma; HLMD-Me-13919: grid square H/I7; 1.57 m below to 2.09 m below stratigraphic marker gamma; HLMD-Me-13920: grid square H/I7; 1.63 m below stratigraphic marker gamma (marked in Fig. 1 with red dots).

*Derivation of epithet:* From the Latin words “*brevis*” (short) and “*rostrum*” (beak) referring to the distinctively short rostrum of this species in comparison to its congeners.

*Diagnosis:* Small shrimp with a short dorsally serrate rostrum and long second pereiopods with strong chela.

*Description:* Small sized shrimp (Figs. 2, 3, 4, 5, 6), total body length 14–19 mm, carapace post-orbital length 5.0–8.5 mm, maximum length about 1.6 of maximum height, laterally compressed, dorsal margin straight, ventral and posterior margin both smooth and convex, no spines discernable besides antennal spine in one paratype (HLMD-Me-13919; Figs. 4F, 6B). Rostrum (Figs. 3, 4F, 7B) short, about one fifth of carapace length, straight, laterally compressed, with an acute distal end, bearing 6–8 spines of equal size on dorsal margin all placed distally to the post-orbital margin and one tooth on ventral margin. Eyes developed, with a globular cornea, broader than eyestalk. Antennules seemingly biflagellate, antennular peduncle about half as long as carapace length. Antennae long, basal segments shorter than the antennular peduncle, with a well-developed scaphocerite about 4 times as long as broad. Left mandible preserved in the holotype (Fig. 7A), incisor process well developed, with three strong teeth, reduced molar process, no evidence of a palp being present. Pereiopods long and slender, first two pairs chelate. Chela of first pereiopod rounded, about three times as long as high, with sharp dactylus twice as long as its maximum height, about the same length as the palmar portion. Second pereiopod much longer and bigger than first, chela about four times as long as high, shorter than carpus, dactylus slightly shorter than palmar portion. Possible sexual dimorphism, with males having longer second pereiopods than females (see remarks below). Last three pairs of pereiopods similar in length and shape. Pleopods poorly preserved. Abdomen smooth, six-segmented, somites with a convex dorsal margin, pleura well developed, first somite reduced, second pleura overlapping both first and third, fourth and fifth somites smallest, similar in shape and size, sixth somite longest. Long telson, about half of carapace length, slightly shorter than uropods. Uropods flabellate, exopod about the same length as endopod, with no diaeresis discernable.

*Remarks*: Internal organs are visible in at least four of the specimens (holotype SF-MeI 5933 and paratypes SF-MeI 13611, HLMD-Me 13919 and HLMD-Me 13920), the holotype being the one with the most of its anatomy preserved (Figs. 2, 3). Just above the mandible (Fig. 7A) is a chitinous structure identified as the stomach (Fig. 9A). Near its posterior end, three hard smaller structures seemingly distinct from the stomach cuticle could be identified with some doubt as gastric ossicles, the largest being likely the mesocardiac ossicle, the one in the middle a zygocardiac ossicle and the bottom one a pterocardiac ossicle. The discovery of new fossils with a similar state of preservation could confirm the presence of such ossicles in this species. Directly posterior to the stomach, a large dark patch is interpreted as the mature ovary, allowing to identify the holotype as an adult female specimen. Near the dorsal margin of the third somite of the holotype, a portion of the intestine has been preserved (Fig. 9B) with two dark pellets interpreted as feces. In the specimen HLMD-Me 13919, the four last pleurobranchiae are preserved (Figs. 4F, 6B).

**Figure 10:**
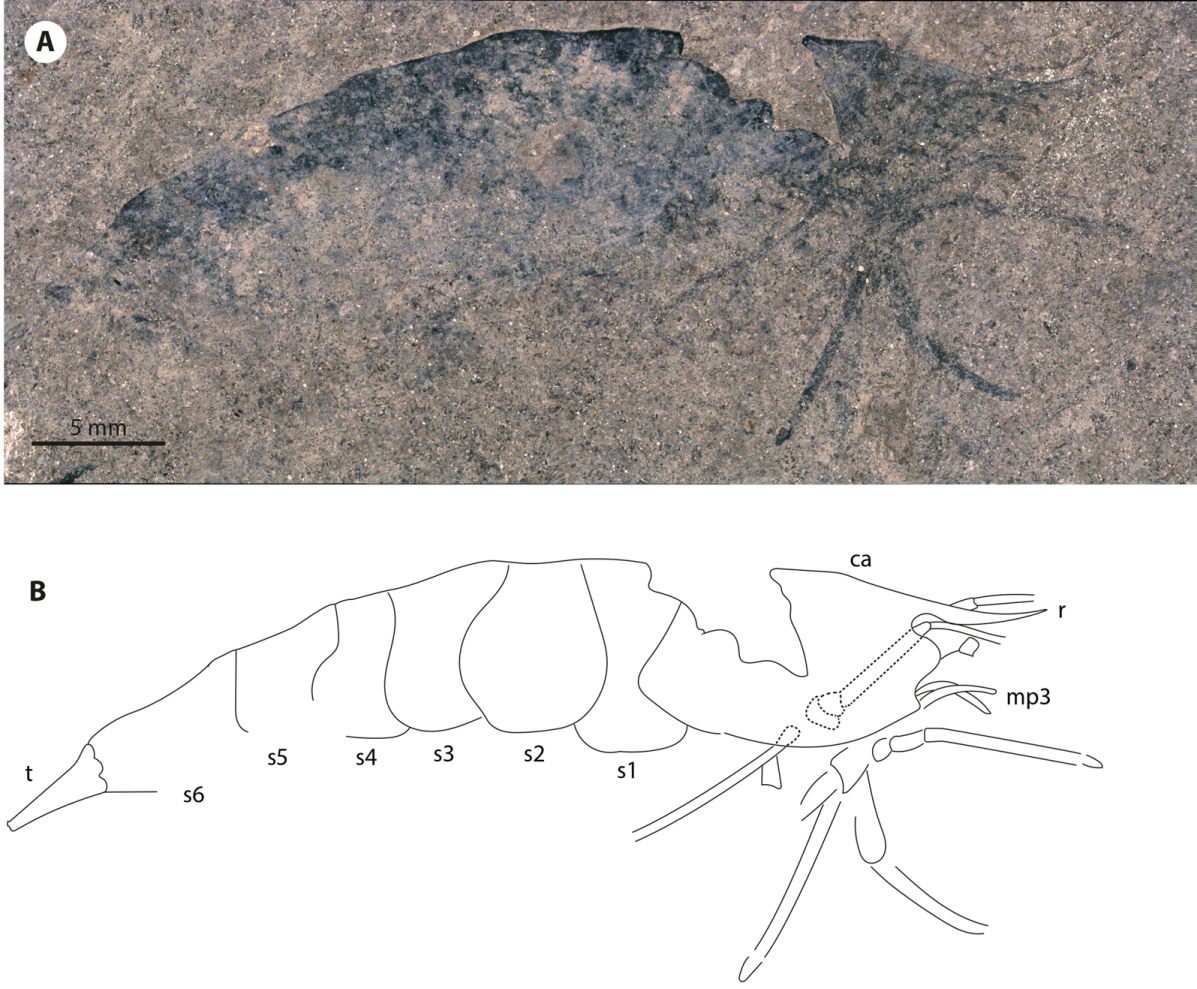
Unidentified shrimp possibly belonging to a different species, HLMD-Me 10646: (**A**) Photograph; (**B**) interpretative drawing. Abbreviations of morphological characters same as in Figs. 3 and 4.

An additional specimen (HLMD-Me 10646: grid square H/I7; 2.80 m below stratigraphic marker gamma; Fig. 10) shows a different morphology with a seemingly long upcurved rostrum which could suggest that it belongs to a different species. It is thus excluded from the paratypes of *B. brevirostris* n. sp. Due to the absence of several important characters in that fossil, we deem it safer to wait until more specimens are discovered before describing it as a new species.

## Discussion

### Systematic placement

There is little doubt about the higher systematic position of these fossils: the second pleura overlapping both the first and third ones, as well as the first two pairs of pereiopods being chelate are characteristic of the infraorder Caridea, and the second pereiopod being larger than the first is a characteristic of the superfamily Palaemonoidea. However, the placement at family level is more difficult, since all the four extant families of Palaemonoidea occur in freshwater: the monogeneric families Desmocarididae and Typhlocarididae, respectively represented by two species of the genus *Desmocaris* from West Africa and four species of the genus *Typhlocaris* from subterranean waters of the Mediterranean region; the Euryrhynchidae, with eleven species in four genera probably from Gondwanan origin (extant species found in South America, Africa and India); and finally the Palaemonidae, by far the largest with more than 276 extant freshwater species and a worldwide distribution^37^. The level of details preserved on the present specimens does not allow to draw any definitive conclusion regarding its placement in one of these four families and they may also belong to an extinct related family. We decided to follow the conservative approach of including the new species in the genus *Bechleja*, maintained with some doubt within the family Palaemonidae. Indeed, the morphology of our fossils fits well with the diagnosis of that genus^35^ and it is a typical shrimp genus of Cenozoic freshwater deposits^38^. There are currently four species in addition to this new one^39^: *Bechleja rostrata* Feldmann, Grande, Birkheimer, Hannibal, and McCoy, 1981 from the Eocene of the Green River Formation, USA, *B. inopinata* Houša, 1957 from the Oligocene of Czech Republic, *B. bahiaensis* Beurlen, 1950 and *B. robusta* Martins-Neto & Mezzalira, 1991, both from the Oligocene of Brazil.

### Taphonomy and preservation of internal organs

Taphonomy experiments on extant shrimps have shown that preservation of shrimp corpses is very sensitive to disturbance and bioturbation^40^ and that soft tissues decay very quickly in normal conditions^41^. This explains the rarity of shrimps in the fossil record and it confirms that the conditions which formed the Messel oil-shale correspond to a meromictic lake stage with permanently anoxic bottom water conditions^17,18^ where the dead shrimps were buried with no bioturbation of the sediment and in the absence of scavengers.

The observations of preserved internal tissues are extremely rare among crustacean fossils and usually require using special imaging methods such as X-ray tomography^42,43^ or auto-fluorescence^44^. The exceptional case of the specimen chosen as holotype (SF-MeI 5933) in which several internal organs can be identified, must be explained by the notably thin exoskeleton of caridean shrimps^45^ coupled with the dissolution of calcium carbonate in Messel sediments^46^. Interestingly, the eyes are preserved on four specimens and appear in the form of white and grey crystals in three of them (Fig. 8).

The mandible being preserved on specimen SF-MeI 5933 (Fig. 9) will be useful for later studies, since it bears important characters to reconstruct the evolution of carideans^47^ and in palaemonoid shrimps in particular^48^. In the same way, the presence of a gastric mill (ossicles in the stomach) is considered a plesiomorphic trait in caridean shrimps and it is usually reduced in extant palaemonids^49^. Its possible presence in our new species may be related to the reduced molar process of the mandible, with mastication taking place predominantly in the stomach rather than the mouth.

### Paleoenvironmental and paleobiogeographical implications

Extant freshwater shrimps of the family Palaemonoidea are usually opportunistic in their diet, being mostly scavengers or detritivores, feeding on plant and animal matter^50^ or sometimes preying upon smaller vertebrates and invertebrates^51^. Even though some organic matter is preserved in the intestine of the holotype, no recognizable element is visible to allow drawing conclusions regarding the diet of these shrimps. The preserved mandible having a small molar process suggests that the fossil shrimps from Messel did not need to crush and grind tough elements but mostly sliced or teared softer matter such as algae strings with their well-developed incisor process^52^.

Rostrum length is often linked to environmental factors in extant freshwater shrimps, with individuals with a short rostrum being found in strong hydrological current conditions whereas individuals with a long rostrum occur mostly in lentic habitats, such as estuaries or lakes^53,54^. It is quite surprising that this new species has a short rostrum given the lacustrine environment where a long rostrum would be expected. This could suggest that the shrimps lived in a tributary instead of the former lake itself, explaining why they are so rare.

Extant related freshwater shrimps mainly occur in tropical and subtropical regions where they inhabit shallow areas of rivers and lakes, living a rather benthic lifestyle among submerged vegetation. Since the fossiliferous oil-shales represent only the deepest part of Lake Messel while the complete shore area is missing, this partly explains their rarity among arthropod fossils from this site, but the reason why the shrimps were found in the middle of the lake remains enigmatic.

Many species of freshwater palaemonid shrimps are currently amphidromous, meaning that they live their adult life in freshwater but the larvae need seawater to develop^55^. This particular life cycle is thought to be a plesiomorphic state which can be lost secondarily in species that become land-locked (i.e. can complete their lifecycle entirely in freshwater)^56^. The presence of rare freshwater palaemonoid shrimps in Lake Messel might suggest a connection to the sea through an outlet flowing to the ocean. The discovery of the single specimen of the fossil eel *Anguilla ignota* Micklich, 1985 supports this hypothesis, as eels also need to migrate from freshwater to the sea and back.

The related fossil species *Bechleja rostrata* Feldmann et al., 1981 is characterized by a rostrum that is longer than the carapace length (vs 0.2 of carapace length in *B. brevirostris* n. sp.). This character agrees well with the lacustrine setup of the Green River’s Fossil Lake, which had an abundant shallow-water benthic fauna^57^ in contrast to our new species. The presence of ostracods in their intestinal tract suggests that decapods of the Green River Formation occasionally fed on them and it is supposed that palaemonids were preyed upon by bowfins^57^. These paleoecological hypotheses can also apply to our species from Messel.

Although the types of lakes are very different, Messel and Green River have many similarities in their fish fauna, as they share genera like the bowfin *Cyclurus*, and the gar genera *Atractosteus* and *Masillosteus*^58^. *Cyclurus* and *Atractosteus* are among the dominant elements of the fish fauna in Messel, while *Masillosteus* is rare in Messel^59,60^. In the Fossil Butte Member of the Green River Formation, all gars are rare, especially the genus *Masillosteus*^58^. Specimens of *Masillosteus* have been found in those sediments of Green River where also shrimps, crayfishes and snails have been found, on which they probably fed^58^, and which might represent nearshore deposits. Especially the occurrence of *Masillosteus* in both Messel and Green River suggests strong paleobiogeographic ties, as might the shared occurrence of the same genus of freshwater shrimp reported in the present study.

Five other species of freshwater carideans have been reported from European Cenozoic deposits (Table 1). *Bechleja brevirostris* n. sp. is the first and only record of freshwater shrimps from the Eocene in Europe and the second worldwide after *Bechleja rostrata* from the Green River Formation (Wyoming, USA). The systematic placement of most of these fossils in the families Palaemonidae and Atyidae remains dubious and would require a thorough re-examination of the specimens.

**Table 1:** Fossil record of freshwater shrimps in Cenozoic deposits of Europe.

Family	Genus	Species	Author, date	Deposit	Country	Geological age
Palaemonidae?	*Homelys*	*minor*	von Meyer, 1862	Öhningen	Germany	Serravallian (Miocene)
Palaemonidae?	*Bechleja*	*inopinata*	Houša, 1957	Kučlin	Czech Rep	Chattian/Aquitanian (Oligocene–Miocene)
Palaemonidae?	*Palaemon*	*exul*	Frič, 1872	Bechlejovice	Czech Rep	Chattian/Aquitanian (Oligocene–Miocene)
Atyidae	*Caridina*	*nitida*	A. Milne-Edwards, 1879	Aix-en-Provence	France	Chattian (Oligocene)
Palaemonidae?	*Micropsalis*	*papyracea*	von Meyer, 1859	Rott	Germany	Rupelian (Oligocene)
Palaemonidae?	*Bechleja*	*brevirostris* n. sp.	Present study	Messel	Germany	Lutetian (Eocene)

It is interesting to note that comparatively to other regions, caridean shrimps are very uncommon in European freshwaters today, mostly represented by a limited number of species in the Mediterranean region with the exception of the atyid *Atyaephyra desmarestii* colonizing waterways up to northern Europe^61^. Anger^62^ suggested a Tethyan origin of the palaemonid genus *Macrobrachium* (to which *Bechleja brevirostris* n. sp. may be related) and explained its absence in Europe today by the Messinian salinity crisis during the Late Miocene that caused estuarine species to disappear from the Mediterranean region. This area could not be recolonized later due to the long distance with the nearest surviving estuarine populations in West Africa^62^. It can be hypothesized that fully freshwater inhabiting species might have survived this period but were soon wiped out by the Pleistocene glaciations and the cooling of the climate in most of Europe. Some southern regions however could have acted as refugia like the Balkans, and the Italian and Iberian peninsulas where some land-locked palaemonid species occur today and where the most diverse fauna of European freshwater shrimps is found^61^.

## Methods

### Illustrations

Specimens from the Senckenberg Forschungsstation Grube Messel were photographed either with a JENOPTIK GRYPHAX camera mounted on a Leica M165 C stereomicroscope associated with PROGRES GRYPHAX software or with a Leica MZ12.5 stereomicroscope with an attached Nikon D300 camera.

Specimens HLMD-Me-10646, 10684, 13919, 13920 were documented using VXH-6000 Digital Microscope. In specimens too large to fit into a single image, different image details in adjacent areas and different focal points were taken and combined into a single stacked and composite image using the microscope’s built-in software. To enhance visibility, we used the built-in function “Shine removal” function.

Photographs at different focal depths were stacked and processed with Adobe Photoshop 2022 and served as basis for digital line drawings made on Adobe Illustrator 2022. All figures were prepared with Adobe Illustrator 2022.

## Data Availability

The specimens described in this study are kept in the collection of the Senckenberg Forschungsinstitut und Naturmuseum Frankfurt/M., located at the Senckenberg Forschungsstation Grube Messel or in the collection of the Hessisches Landesmuseum Darmstadt, and are all available for study upon request. All information relevant for this investigation is presented in this paper.
