# Author Correction: Exceptional preservation of internal organs in a new fossil species of freshwater shrimp (Caridea: Palaemonoidea) from the Eocene of Messel (Germany)

**DOI:** 10.1038/s41598-023-32986-7

**Published:** 2023-04-12

**Authors:** Valentin de Mazancourt, Torsten Wappler, Sonja Wedmann

**Affiliations:** 1 Laboratoire Biologie des Organismes et Écosystèmes Aquatiques MNHN, CNRS 8067, SU, IRD 207, UCN, UA, Muséum National d’Histoire Naturelle, Paris, France; 2 Center for Integrative Biodiversity Discovery, Museum für Naturkunde Leibniz Institute for Evolution and Biodiversity Science, Paris, Germany; 3 Department of Natural History, Hessisches Landesmuseum, Darmstadt, Germany; 4 Section Palaeontology, Institute of Geosciences, Rheinische Friedrich-Wilhelms-Universität Bonn, Bonn, Germany; 5 Senckenberg Forschungsstation Grube Messel, Senckenberg Forschungsinstitut und Naturmuseum Frankfurt/M., Messel, Germany

Correction to: *Scientific Reports* https://doi.org/10.1038/s41598-022-23125-9, published online 27 October 2022

de Mazancourt et al. (2022) described a new species of fossil freshwater shrimp, *Bechleja brevirostris* from the Eocene of Messel (Germany). Although the species is fully characterized and figured in the original description, it was published in an online-only journal issue and the article does not include evidence of registration in ZooBank within the work itself, which is a requirement by Article 8.5.3 of the International Code of Zoological Nomenclature^1^. Therefore, the newly proposed species-group name *Bechleja brevirostris* is not available.

The present publication has been registered in ZooBank with the LSID: urn:lsid:zoobank.org:act:D9D7741A-AD8A-48B1-B6E6-B720C376307B. The following ‘Systematic Paleontology’ section modified from the original article^2^ appears below. In addition, the collection data of the holotype is corrected in the text and in Fig. 1, since the information provided in the original article revealed to be erroneous. The correct Fig. 1 and accompanying legend appear below.

**Figure 1:**
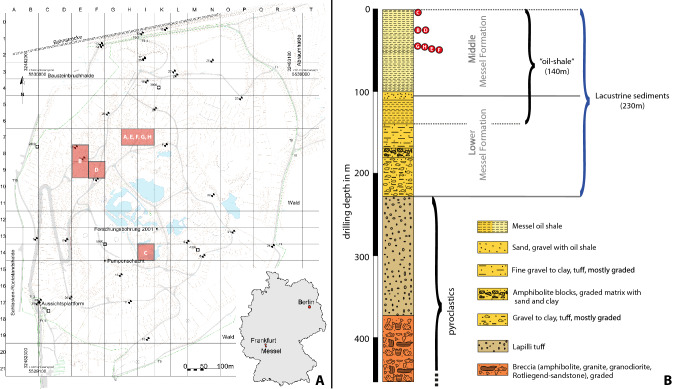
(**A**) Map of the Grube Messel site (from^12^) with the location of each shrimp fossil (**A**–**H**) indicated. (**A**) SF-MeI 5933 (holotype); (**B**) SF-MeI 13611; (**C**) SF-MeI 14640; (**D**) SF-MeI 16018; (**E**) HLMD-Me-10684; (**F**) HLMD-Me-10646; (**G**) HLMD-Me-13919 and (**H**) HLMD-Me-13920. (**B**) Section of the Grube Messel core (modified from^13^). Red circles indicate the corresponding layers where the fossil shrimps were found. Depth: for (**C**) ca. 2.96m to 2.16m; for (**B**) and (**D**): ca. 24.86m to 23.86m; for (**A**): ca. 27.46m to 27.06m; for (**G**): ca. 46.49m to 45.97m; for (**H**): ca. 46.03m; for (**E**): ca. 46.26m; for (**F**): ca. 47.2m.

***Systematic paleontology***

**Order Decapoda Latreille, 1802**

**Infraorder Caridea Dana, 1852**

**Superfamily Palaemonoidea Rafinesque, 1815**

**? Family Palaemonidae Rafinesque, 1815**

**Genus Bechleja Houša, 1957**

***Bechleja brevirostris*** **n. sp.**

ZooBank LSID: urn:lsid:zoobank.org:act:D9D7741A-AD8A-48B1-B6E6-B720C376307B.

*Bechleja brevirostris* de Mazancourt, Wappler & Wedmann, 2022: figs. 2–9.

*Type material:* SF-MeI 5933, holotype, plate (A) and counterplate (B), Mrs Christa Behnke leg.; SF-MeI 13611, plate (A) and counterplate (B); SF-MeI14640, plate (A) and counterplate (B), SF-MeI 16018, plate (A) and counterplate (B), HLMD-Me 10684, HLMD-Me 13919, HLMD-Me 13920, paratypes.

*Type locality*: Grube Messel, near Darmstadt, Hesse, Germany (Fig. 1).

*Stratigraphic information*: Holotype SF-MeI 5933: no data; SF-MeI 13611: grid square E8/9; 2.5m above to 3.5m above local stratigraphic marker level alpha; SF-MeI 14640: grid square i14; 0.95m above to 1.75m above local stratigraphic marker level M; SF-MeI 16018: grid square F9; 2.5m above to 3.5m above local stratigraphic marker level alpha; HLMD-Me-10684: grid square H/I7; 1.86m below stratigraphic marker gamma; HLMD-Me-13919: grid square H/I7; 1.57m below to 2.09m below stratigraphic marker gamma; HLMD-Me-13920: grid square H/I7; 1.63m below stratigraphic marker gamma (marked in Fig. 1 with red dots).

*Derivation of epithet:* From the Latin words “*brevis*” (short) and “*rostrum*” (beak) referring to the distinctively short rostrum of this species in comparison to its congeners.

*Diagnosis:* Small shrimp with a short dorsally serrate rostrum and long second pereiopods with strong chela.

*Description:* Small sized shrimp, total body length 14–19mm, carapace post-orbital length 5.0–8.5mm, maximum length about 1.6 of maximum height, laterally compressed, dorsal margin straight, ventral and posterior margin both smooth and convex, no spines discernable besides antennal spine in one paratype (HLMD-Me-13919). Rostrum short, about one fifth of carapace length, straight, laterally compressed, with an acute distal end, bearing 6–8 spines of equal size on dorsal margin all placed distally to the post-orbital margin and one tooth on ventral margin. Eyes developed, with a globular cornea, broader than eyestalk. Antennules seemingly biflagellate, antennular peduncle about half as long as carapace length. Antennae long, basal segments shorter than the antennular peduncle, with a well-developed scaphocerite about 4 times as long as broad. Left mandible preserved in the holotype, incisor process well developed, with three strong teeth, reduced molar process, no evidence of a palp being present. Pereiopods long and slender, first two pairs chelate. Chela of first pereiopod rounded, about three times as long as high, with sharp dactylus twice as long as its maximum height, about the same length as the palmar portion. Second pereiopod much longer and bigger than first, chela about four times as long as high, shorter than carpus, dactylus slightly shorter than palmar portion. Possible sexual dimorphism, with males having longer second pereiopods than females (see remarks below). Last three pairs of pereiopods similar in length and shape. Pleopods poorly preserved. Abdomen smooth, six-segmented, somites with a convex dorsal margin, pleura well developed, first somite reduced, second pleura overlapping both first and third, fourth and fifth somites smallest, similar in shape and size, sixth somite longest. Long telson, about half of carapace length, slightly shorter than uropods. Uropods flabellate, exopod about the same length as endopod, with no diaeresis discernable.
